# Supplementary material for: Are carbon emissions trading and green financial instruments synergistic? -Comprehensive quantitative research based on content analysis
Source: PLoS One. 2024 Mar 7;19(3):e0298601. doi: 10.1371/journal.pone.0298601 (PMC10919601; doi:10.1371/journal.pone.0298601)
Supplement: S1 Data — (ZIP) [file pone.0298601.s001.zip › Data/地级市Z_F工作报告.zip!/─■╧─/╓╨╬└╩╨/└┤╫╘╣1⁄2╓┌║┼í╛┬φ┐╦╩2╛▌═°í┐_2008.docx]

各位代表：

现在，我代表市人民政府向大会作政府工作报告，请予审议，并请市政协委员和其他列席人员提出意见。

新市建设取得巨大成就

2004年2月12日，中卫市成立。四年来，我们在党中央、国务院的亲切关怀下，在自治区党委、政府和市委的正确领导下，坚持以邓小平理论和“三个代表”重要思想为指导，全面贯彻落实科学发展观，认真贯彻落实自治区第九次和第十次党代会精神，扎实苦干，爬坡追赶，着力推进又好又快发展，大力促进社会和谐，圆满完成了既定目标和任务。一个新市行政机构已经诞生，一个新市行政区划全面完成，一个新市城市框架完全拉开，一个新市国民经济基础初步奠定。总之，经过四年奋斗，一个禀赋独特、充满魅力、生机勃发、奋力追赶的新中卫展现在世人面前。

经济实力大幅提升。预计2007年，实现地区生产总值88.6亿元，年均递增11.6%。其中：第一产业16.7亿元，年均递增4.6%；第二产业34.4亿元，年均递增16.8%；第三产业37.5亿元，年均递增11.1%。人均生产总值8461元，年均递增9.8%。预计完成财政总收入5.69亿元，年均递增22.8%；其中市本级地方财政收入完成3.95亿元，年均递增31.2%。工业经济快速发展。预计2007年实现工业增加值26.5亿元，是2003年的2.3倍，比2006年增长23.6%，年均增长19.6%。工业化步伐加快，造纸、酿酒、冶金化工、建筑建材、农副产品加工、机械制造等优势特色产业进一步壮大。打造工业发展基地平台，开工建设了中卫美利、中宁宁新2个工业园区和镇罗金鑫园、常乐陶瓷园、宁夏红科技园等6个专业园区。全市累计完成工业项目投资85亿元，培育壮大年销售收入过10亿元的企业1家，过亿元的22家，过5000万元的31家。电网基础设施建设进一步完善。万元地区生产总值能耗比2006年下降1%。特色农业优势凸显。压砂面积由设市前的9.8万亩增加到101.3万亩，硒砂瓜产业成为中部干旱带农民增收的一项主要产业，香山硒砂瓜成为中卫走向全国的一张“绿色名片”；设施蔬菜、枸杞分别达到11.9万亩、21.5万亩，分别比2003年增加9.3万亩、12.2万亩；优质饲草面积巩固在120万亩，经果林面积达到37.2万亩；马铃薯、优质米、小茴香种植面积分别达到84.3万亩、17.2万亩、10.1万亩。中宁枸杞、香山硒砂瓜、设施蔬菜成为全国绿色食品原料标准化生产基地。鸡产业得到恢复性发展，饲养量达到600万只。农业产业化步伐加快，新建现代工厂化育苗中心，全市各类农产品加工企业发展到160家，农民专业合作组织和农产品流通协会达到176个。农业优势特色产业已成为拉动农民增收的支柱产业。旅游业带动作用明显增强。累计完成旅游项目投资2.2亿元，形成了一批特色鲜明的旅游精品。沙坡头成为国家首批5A级旅游景区。成功举办了大漠黄河国际旅游节、法国人“丝绸之路”长跑活动、香山硒砂瓜节、中宁枸杞节、海原花儿艺术节等节事活动，旅游知名度进一步提高。全市旅游人数、旅游总收入年均递增16.5%和33.6%。商业、服务业、餐饮娱乐业等第三产业迅速发展。社会消费品零售总额达到18.8亿元，年均递增14.4%。

城乡面貌日新月异。四年来，全社会固定资产投资累计完成244.1亿元，年均递增12.8%。建成了一批重要的能源、交通、水利和城市公共基础设施。城市建设快速推进。改造提升老城区，加快建设城市新区。建成中央大道、迎宾大道等9条80公里城市主干道路，改造老城区“八街十二巷”，市区形成“八横十纵”的路网体系。启动建设中卫——中宁滨河大道工程。建设黄河公园、香山湖、生态公园、景观水道等一大批城市公用基础设施项目，形成水面4500亩。开工建设行政中心等24个单位办公楼，建筑面积22万平方米。城市建成区面积由12平方公里拓展到32平方公里，绿地面积由59万平方米增加到463万平方米。城区进入“中国特色魅力城市200强”。中宁县裕民街、团结路等17条主次干道和世纪花园、行政中心等140个项目投入使用；海原县集中供水供热、垃圾处理、巷道硬化、街道改造等81个项目全面完成。城镇服务功能日趋完善，以市区为中心，中宁、海原为骨干，中心镇为基础的城市体系初步形成，城镇化率由2004年的25%提高到2007年的32%。基础设施建设步伐加快。建设硒砂瓜补灌工程，控灌面积达到50万亩。实施“塞上农民新居”和山区危窑危房改造等农村公共事业项目，启动农村新能源沼气工程，2.2万户农民用上了沼气、太阳灶。建成新农村示范点41个，2.8万户农民彻底告别了危窑、土坯房，迁入新居。建成中卫汽车客运总站。硬化乡村主干道路816.6公里。全市公路通车总里程达4895公里，实现了乡乡通油路、村村通公路。中营高速公路中卫段建成通车，实现了两县一区1小时内上高速公路。沙坡头机场航站楼和跑道工程开工建设。资源与环境保护全面加强。连续四年实现耕地占补平衡。退耕还林、退牧还草和封山禁牧成效显著，森林覆盖率达到13.9%，沙漠化和水土流失得到控制。重点流域、重点区域和城市环境质量明显改善，主要污染物排放量逐年下降。

改革开放扎实推进。严格兑现粮食直补政策，提前一年取消了农业税及其附加。中宁县和山区乡镇债务全部化解。水权转换改革有序推进。企业改革继续深化，城区破产企业退休人员和失业人员基本医疗、养老保险遗留问题得到有效解决。国企所属中小学和医院移交地方管理。机关事业单位工资制度改革基本完成。地方金融机构监管工作得到加强。“乡财县管乡用”、政府收支分类、国库集中支付等改革全面推行，公共财政体系建设步伐加快。投资体制改革逐步完善，非经营性政府投资项目“代建制”试点启动实施。对外开放不断扩大。全市累计引进各类招商引资项目504个，实际到位资金72.7亿元。成功引进中国冶金科工集团重组美利纸业，使其跨入全国造纸行业前列；引进电解铜和单晶硅项目，填补了宁夏有色金属和太阳能级单晶硅生产空白；引进科豪、美康等陶瓷企业，生产能力达到3500万平方米；与中电投集团黄河上游公司、江苏扬农化集团达成战略合作意向。对外贸易逐年扩大，累计进出口总额达到1.2亿美元，其中出口9600万美元，比2004年增长69%。

人民得到实惠更多。城镇居民人均可支配收入和农民人均纯收入预计分别达到9245元和3060元，年均递增11.7%和9.1%；城乡居民存款余额达到53.4亿元，年均递增7.5%。新增就业2.8万人，城镇登记失业率控制在3.7%以内。农村富余劳动力转移就业净增18.6万人，清理拖欠农民工工资4349.3万元。加快建立覆盖城乡的社保体系，财政四年累计投入1.3亿元。基本养老、医疗、失业、工伤等保险人数分别增长8%、20.4%、52.6%和76.5%，2007年享受最低生活保障的城乡居民达到8.5万人。住房公积金制度进一步完善。累计投入救灾救济资金7489万元，救济受灾群众56.7万人次，解决了32.8万农村人口饮水困难，保证了受灾群众有粮吃、有水喝。组织输出务工人员51.5万人次，实现劳务收入16.1亿元，其中海原县达37万人次，收入12.7亿元。第一批27个村“整村推进”扶贫开发工程全面完成，绝对贫困人口由5.9万人减少到3.8万人。应对突发公共事件、防灾减灾、重大疫情处置体系初步建立。安全生产监管进一步加强，依法取缔了水上违法船运，启动了禁止农用车载人实施方案，全市安全生产形势基本平稳并趋于好转。产品质量和食品药品安全水平不断提高。

社会事业快速发展。制定实施“十一五”科学技术发展规划等一系列重大政策，取得自治区级重大科技成果32项，新建企业技术中心3个，大河机床数控珩磨技术达到国际先进水平。科技特派员创业行动不断深化，科技创新能力和全民科学素质得到提高。中卫城区成为宁夏第一个国家可持续发展实验区。素质教育全面推进，全部免除了义务教育阶段学杂费，海原县提前一年实现“两基”攻坚目标。适龄儿童入学率由98.3%提高到99.8%，高中毛入学率由46.7%提高到57.7%。中等职业教育在校生达到3707人。高等教育毛入学率达到52.8%。民族教育发展步伐加快，少数民族在校生比例与人口比例基本相当。新建中卫一中、中卫中学（香山回民中学）、中卫六小、中宁一中、海原一中等中小学。创办《中卫日报》，实施广播电视“村村通”工程，广播、电视覆盖率均达到96%。“三下乡”活动扎实推进，广场文化演出594场（次），群众文化生活进一步丰富。非物质文化遗产保护进一步加强。成功举办全国首届大漠健身运动会等赛事活动。深入实施“体育下乡、篮球进村”工程。建成五环广场、体育训练馆等一批体育基础设施，全民健身服务体系日益完善。医疗卫生事业迈上新台阶。公共卫生体系、社区卫生服务体系及县、乡、村卫生服务网络逐步完善，人民群众医疗保障水平明显提高。实施新型农村合作医疗制度，群众看病难、看病贵问题有所缓解。成功防控高致病性禽流感，重大疫情防范能力不断提高。“少生快富”工程和奖励扶助制度全面推进，人口自然增长率下降到9.75‰。群众性精神文明创建活动深入开展，国防动员和双拥共建进一步加强。审计、统计、气象等工作取得新成绩。外事侨务、妇女儿童、老龄、残疾人等事业健康发展。

民主法制建设不断加强。自觉接受人大代表和政协委员监督，四年共办理自治区、市人大代表议案建议146件、政协提案667件，办复率100%。全面落实《依法行政实施纲要》，削减行政审批事项14项。认真贯彻《公务员法》，公务员队伍建设得到加强。建立完善《中卫市人民政府督察督办制度》等七项制度，开通市长信箱，建立市级领导和部门负责人信访接待日制度，群众的一些热点难点问题得到有效解决。开展商业贿赂专项治理，强化审计监督，反腐倡廉取得积极成效。稳步推进社区建设和村民自治，群众的民主权利得到保障。认真贯彻党的民族宗教政策，依法加强宗教事务管理，各民族共同团结奋斗、共同繁荣发展的良好局面得到巩固和发展。深入开展普法教育，切实加强社会治安综合治理，“平安中卫”取得实效。

各位代表，回顾四年不平凡的历程，最显著的变化是经济社会发展跃上新台阶，蓄势待发的态势正在显现；最可喜的变化是围绕又好又快、以川济山、以城带乡、快速发展的局面已经形成；最深刻的变化是领导苦抓、干部苦帮、群众苦干，激情创业、热情干事的氛围更加浓厚。这些成绩和变化，是中央、自治区和市委正确领导的结果，是市人大、政协和社会各界大力支持的结果，凝聚着全市干部群众智慧、心血和汗水。在此，我谨代表市人民政府，向全市人民，向辛勤工作在各条战线上的工人、农民、知识分子和干部，向驻卫部队指战员和武警官兵、公安司法干警，向各民主党派、各人民团体和各界人士致以崇高的敬意！向中央、区属驻卫单位，向关心支持中卫现代化建设的各界朋友，向来中卫创业发展的各位企业家和建设者表示衷心的感谢！

我们也清醒地看到，经济社会发展中的制约和问题还不少。一是解放思想还不够，创新体制机制还不能适应发展社会主义市场经济需求。二是经济总量小，人均地区生产总值仅是全区的62%、全国的45.7%。表现为：农业现代化水平低，特色农业比重小；工业基础薄弱，门类单一，结构不够合理，增长方式粗放，高投入、高耗能、高排放、低效益的问题比较突出；服务业还处于较低层次。三是城镇化水平低，城市聚集辐射带动作用还不明显。四是城乡居民收入与全国平均水平差距还较大，特别是农民持续增收难度加大。五是财政自给率低，收支矛盾突出。六是社会事业发展滞后，综合性文体设施还不健全，城乡就业和社会保障仍面临较大压力。七是个别地方和政府部门执行效率不高，浪费和腐败现象还不同程度地存在。对这些问题，我们将认真研究，采取有效措施，切实加以解决。

跨越式发展的新目标新任务

党的十七大提出了人均国内生产总值到2020年翻两番的宏伟目标，自治区第十次党代会提出了跨越式发展的新要求，这为我们今后五年的发展指明了前进方向，吹响了进军号角。实现全面小康，全国经济总量年递增只需7%，全区需11%，而我们必须保持在13%以上，任务光荣而艰巨。面对中央和自治区新的要求，面对105万人民对美好生活的期盼，我们必须实现跨越式发展！面对东部率先发展、中部迅速崛起、西部竞相追赶的严峻形势，我们必须实现跨越式发展！这是时代赋予我们的历史使命，我们必须向全市人民交上一份跨越式发展的满意答卷！

随着经济全球化、产业梯度转移和西部大开发的深入，我们迎来了跨越式发展的难得机遇和广阔空间。水利、电力、交通和土地等资源，为我们跨越发展创造了比较优势；四年所取得的巨大成就，为我们跨越发展奠定了坚实基础；全市上下人心思进、爬坡追赶的强烈愿望，为我们跨越发展提供了精神动力。我们有决心、有能力与全市人民一道，开创新局面、取得新业绩、实现新跨越。今后五年的总体要求是：高举中国特色社会主义伟大旗帜，以邓小平理论和“三个代表”重要思想为指导，深入贯彻落实科学发展观，全面贯彻党的十七大、自治区第十次党代会和市第二次党代会精神，以跨越式发展为主题，围绕“六大目标、九大任务”，着力推进新型工业化、农业现代化、城市精品化、旅游特色化进程，加快发展各项社会事业，切实改善人民生活，努力提高政府行政效能，为建设经济繁荣、环境优美、社会和谐、人民富裕的新中卫而努力奋斗！

六大目标是：

——经济总量跨越180亿元大关。地区生产总值翻番，年均增长13%以上，人均达到16070元，与全区的差距从38%缩小到19.6%。地方财政一般预算收入翻番，达到7.4亿元，年均增长14.6%。全社会固定资产投资翻番，达到150亿元，年均增长16%。工业增加值翻番，达到76亿元，年均增长20%。

——经济发展方式实现重大转变。新型工业化步伐加快，建立循环经济工业园区，节能减排效果显著，优势特色产业群基本形成，引入培育3到5个产值在50亿元以上的骨干企业，工业增加值占gdp的比重达到40%以上。万元gdp综合能耗五年降低20%，年均下降4%；二氧化硫和化学需氧量排放五年分别降低18%和14.2%。现代农业扎实推进，土地产出率、农产品加工转化率和商品率、劳动生产率有较大提高，农民收入增长较快。

——人居环境明显改善。城镇化率达到45%以上；市区进入国家卫生城市、国家园林城市、国家环保模范城市、中国优秀旅游城市行列。全市森林覆盖率达到18%；城市绿地率达到25.9%以上；市区绿化覆盖率达到38%以上；集中供热普及率90%以上，生活垃圾无害化和污水处理率均超过93%。中宁县达到自治区级园林城市和卫生城市标准。农村环境明显改善，村庄绿化、巷道硬化、环境美化。

——社会事业取得新发展。基本普及高中教育，高中毛入学率达到95%；大力发展职业教育，职业教育占高中教育的53%。建成体育馆、沙漠治理博物馆、影剧院、文体艺术中心、体育场、老干部活动中心等一批文化体育设施。医疗、养老、救济、大病救助等社会保障体系基本健全。城镇每年新增就业岗位8000个，城镇登记失业率控制在4.5%以内；农村劳动力转移就业规模稳定在18万人，零就业家庭每年至少有一人实现就业。

——人民生活水平有新提高。社会消费品零售总额达到40亿元，年均增长16%；物价保持基本稳定；城镇居民人均可支配收入达到16300元，年均增长12%；农民人均纯收入达到4500元，年均增长8%。其中，海原农民人均纯收入达到2900元，年均增长10%。人口自然增长率控制在12‰以内。

——山川协调发展取得根本突破。在国家、自治区对海原县功能区划调整、8万人移民工程和全面职业教育有力推动下，培育海原县新的经济增长极，从根本上解决影响海原发展的制约问题，使海原经济社会走上可持续发展之路。

九大任务是：

一、着力提升工业生产能力，在推进新型工业化上取得新突破

打造工业发展平台。举全市之力建设“一区多园”。把美利工业区作为工业强市的“一号工程”，在资金、项目上集中安排，加快完善水、电、路、通讯、环保、绿化等基础设施，重点建设工业污水处理湿地和现代物流园区，配套建设6000亩的城市森林公园，用十年时间建成占地25平方公里、环境优美、集约发展的工业城。加快建设中宁工业园和常乐陶瓷园、镇罗金鑫园、宣和高载能园、宁夏红科技园、迎水桥精细化工园，形成门类齐全、布局合理、结构优化的工业发展格局。

培育壮大优势特色产业集群。以循环经济的理念，依托区位、资源、基础和特色优势，培育一批带动力强的大企业、大集团。集中力量扶持壮大造纸、冶金化工、新型建材、农副产品加工、机械制造五大优势产业集群。重点抓好以中冶美利纸业集团为龙头的造纸业，加快林纸一体化项目建设，形成100万亩速生林、100万吨造纸和60万吨纸浆生产能力的现代化造纸产业集群；建设一批电石、pvc、钢铁等高载能项目，实现年产电石、pvc、钢铁“三个百万吨”的目标，形成以pvc及下游产品、热电联产为主的冶金化工产业集群；建设镁及镁合金、碳化硅、单晶硅、多晶硅、陶瓷、石膏等重点项目，形成新型建材产业集群；以宁夏红集团为龙头，发展枸杞酒酿造业，打造中国枸杞第一品牌，争取宁夏红集团二板上市，形成以枸杞制品等为主的特色农产品加工业集群；建设千台机床、万吨以上铸件等重点项目，发展壮大以数控机床为主导的先进装备制造业集群。全市规模以上企业发展到120家以上，年实现工业总产值220亿元，工业增加值66亿元。

促进个体、私营经济和中小企业发展。认真贯彻落实《中小企业促进法》，推进公平准入，改善融资条件，破除体制障碍，加强协调服务，为个体、私营经济和中小企业发展创造更加良好的环境。鼓励具备条件的企业上市。积极营造鼓励银行信贷投入的政策环境，建立中小企业贷款担保体系，出台中小企业贴息办法，大力扶持发展科技型、外向型、资源深加工型、配套服务型、劳动密集型的个体、私营经济和中小企业，为地方经济发展和解决就业作出应有的贡献。积极创建名牌产品、驰名商标。

抓好技术进步和节能减排。以信息化带动工业化，围绕五大优势特色产业关键领域和重点环节，实施20项重大产业技术开发、升级和创新项目，确保每年的技改投资增长20%以上，在今后五年中完成新一轮技术改造。严格实行节能减排，抓好重点耗能企业节能降耗，坚持上大压小，淘汰落后产能，每个工业园区均按环保标准控制排放。新建和重组工业项目全部进入工业园区，走出一条低消耗、低排放、高效益的新型工业化路子。

大力发展“引入经济”。紧紧抓住我国经济由东向西梯度推进、世界性产业转移由我国沿海向内地延伸的重要战略机遇，坚持本地发展与外部引进并重的原则，大力发展“引入经济”，促进跨越式发展。营造优良承接环境，降低投资门槛，做好跟进服务一条龙工作，营造全程、高效、优质的服务机制和优良、宽松的投资环境；打造一流承接平台，以自己最具特色、效益最好、实力最强的企业或项目与转移对口产业合作，积极打造具有本地优势特色的工业园区。瞄准转移产业中相应的大企业、大项目和大资金，切实做好工业园区的各项基础配套工作。把“一区多园”作为系统工程来建设，努力构建一个多行业、多区间和多功能、具有完整产业链的工业园区系统，形成现代化产业生产基地，构筑开放型经济的强力增长极。

二、着力推进农业提质增效，在新农村建设上取得新突破

大力发展现代农业。坚持实施优势特色产业发展战略，突出扩规、提质、增效，做大做强硒砂瓜、设施蔬菜、枸杞、马铃薯、苹果、红枣等特色产业，促进农民增收。到2012年，硒砂瓜种植面积巩固在100万亩，产值达到10亿元以上；新增设施蔬菜18万亩，累计达到30万亩，产值达到20亿元；新增枸杞8.5万亩，累计达到30万亩，产值达到10亿元；建设城区北山生态林带、中营高速公路两侧和南山台子苹果产业带、香山北麓红枣产业带，新增优质苹果15万亩，累计达到27万亩，新增红枣50万亩，累计达到70万亩。继续大力发展饲草、小茴香、马铃薯等山区增收特色产业。重振鸡产业，饲养量达到1000万只，生猪、牛、羊存栏分别达到100万头、30万头、150万只。发展农民专业合作组织，支持农业产业化经营和龙头企业发展。建成2个年流通量30万吨的硒砂瓜专业批发市场和2个年流通量20万吨的蔬菜专业批发市场。加快建设出口农产品基地，培育产值过亿元的龙头企业5家，过5000万元的12家。加快科技推广与服务体系建设，扎实推进科技特派员创业行动和农业科技入户工程，加快新技术、新品种推广转化步伐。加强动植物疫病防控，提高农产品质量安全水平。实施品牌战略，建设6个自治区级“绿色食品”和无公害农产品标准化示范基地，85%的农产品通过无公害、绿色产品认定和认证。

强化农业发展基础。建立“设施农业所有权登记制度”，解决设施农业发展资金不足问题。改革用水制度，加大水权转换力度，大力发展节水农业。探索实行农村土地股份合作，发展多种形式的规模经营。扩大农村政策性保险覆盖面，提高农业防灾抗险能力。严格执行耕地保护制度，确保粮食总产保持稳定。调整投入布局，整合项目资金，确保财政支农投入、固定资产投资逐年增加。完善各项支农惠农政策，扩大粮食、良种、农机购置、旱作农田以及农业生产资料综合补贴的规模和范围，落实退耕还林、退牧还草等各项补助。加强以农田水利建设为主体的农业基础设施建设，加大黄河险工段治理力度，增强农业保障能力。壮大农村集体经济。

改善农村人居环境。加大投入力度，加快基础设施向农村延伸，公共服务向农村覆盖的进程。以“塞上农民新居”、“危窑危房改造”和村容村貌整治为着力点，通过资金、原材料补助等方式，引导农民加快坡屋顶、砖瓦房为主要风格的新民居建设。加强卫生室、农家店、文化室为一体的标准化村部建设。推广沼气、太阳能等清洁能源建设，改善农村卫生环境。围绕主干路两侧建设经果林景观带，绿化、美化村庄，使村容村貌整洁优美，饮用水质达到标准，水源污染得到有效控制。大力发展客运市场，推进农村客运公交化，解决群众出行问题。

三、着力优化城市功能和品位建设，在打造宜居宜商宜游环境上取得新突破

紧紧抓住自治区实施沿黄城市带发展战略的机遇，着力构筑以市区为中心，中宁、海原县城为骨干，小城镇为补充的城镇体系，着力完善功能，提升品质，把中卫建设成为沿黄城市带上最亮丽的城市。

推进城市资本化经营。充分发挥政府投资的导向作用，建立以市场为导向、企业为主体、存量资产为重点的投融资体制。对可经营的重大基础设施、公共建筑项目逐步进行产业化经营，调控社会资金，实现城市建设自我积累、自我发展的良性循环。不断完善城市管理体系，进一步健全土地收储制度，盘活土地资产。加快城市供水、供热、供气等公用事业管理体制改革，多渠道筹措资金，形成多元化投融资、企业化经营、滚动开发的城市资产经营新路子。深化户籍管理制度改革，快速聚集城市人口，发展壮大城市产业。强化城市环境综合治理，实施城市净化、绿化、美化、亮化“四化”工程，改善城市环境卫生，提高城市管理水平，塑造良好的城市形象。

加快城市精品化建设。按照“近水亲河、东扩南移”的思路，深度挖掘中卫历史文化内涵，做好做活黄河水域文章，加快城市建设步伐。完善市区、中宁、海原控制性详规，做好各项专业规划的完善和衔接，注重城市色调与城市风貌的相互协调，充分展示城市个性特色。深度开发利用黄河公园、香山湖、腾格里湖等湿地湖泊，贯通沙坡头大道景观水道，初步建成城市生态水系。加快以公共绿地、环城林带为重点的生态园林建设，精心构建林水相依、品位高雅的园林绿化景观，努力实现“城在水中、园在城中、水景相融”的目标。突出城市的经济、生态和旅游三大功能，加快城市交通、供暖、供水、供电、排污网络建设，完善配套各类基础设施。加快城市公共服务设施建设，打造宜居、宜商、宜游的人居环境、置业环境和经济环境。

促进城乡一体化发展。明晰两县一区整体空间布局和功能定位，做到统一规划、资源共享、设施共建、优势互补、协调发展。以道路、供水、供电、学校等公共设施和基础设施建设为重点，增强城市承载能力，形成区域化的经济、文化中心，辐射带动县域经济发展。以迎水桥、宣和、镇罗、常乐、石空、兴仁为重点，突出集镇供水、污水处理、垃圾处理、文化卫生等服务功能建设，重点发展工矿型、交通枢纽型、商贸型、旅游型的中心小城镇，以业兴镇、以镇聚业，形成工业化、产业化、城镇化互为作用、相互促进的发展新格局，加快城乡一体化进程。

四、着力壮大旅游产业，在培育新的经济增长点上取得新突破

围绕争创中国优秀旅游城市目标，打响“黄河古城、浪漫沙都、花儿杞乡”城市品牌。大力发展特色旅游。着力培育沙坡头景区，重点发展以沙坡头、水稍子、通湖草原为主的沙漠观光旅游，以黄河漂流、沙宁滨河大道景观带为主的黄河观光旅游，以高庙、石空大佛寺为主的寺庙观光旅游，以中宁枸杞、香山硒砂瓜为主的农业观光旅游，以寺口子为主的登山攀岩旅游，以海原花儿为主的回乡风情旅游，以海原环球大地震遗址为主的灾害文化旅游。加快旅游项目建设。规划建设沙坡头南岸景区、海原地震遗址博物馆等重点项目。新建改建一批三星级宾馆。统一旅游标识标牌。加快旅游信息化建设。积极争取国家和自治区资金支持，广泛吸纳社会资本和民间投资，参与旅游开发和旅游项目经营。规范旅游行业管理。加强旅游行业诚信建设，严格旅游执法，严厉打击破坏旅游市场秩序的各种违法违规行为。健全旅游投诉制度，切实维护游客利益。逐步规范导游服务、团队操作、景区管理、商品购物等各个环节，提高旅游景区、宾馆饭店、旅行社等旅游企业的管理水平和服务质量。积极拓展客源市场。巩固西北市场，大力开发长三角、珠三角市场，积极拓展海外市场。进一步加强与周边地区的旅游合作，实现资源共享、互利双赢。每两年举办一次全国大漠黄河旅游节和大漠运动会，办好重大节事活动和旅游推介会，提高旅游知名度，把中卫打造成中国优秀旅游城市、区域性国际旅游目的地，使旅游业成为我市新的经济增长点。

五、着力发展物流产业，在繁荣现代服务业上取得新突破

发展现代物流。积极争取政策扶持，建成中卫保税区，发展路铁联运，形成以市区为中心、以包兰铁路、太中银铁路、中营高速公路为纽带的物流基地发展格局。大力发展交通运输产业和现代物流产业，规划建设一批物流园区和专业市场。加快航空、陆路和电子口岸建设，力争使我市成为西北地区的低成本物流通道。整合本地物流企业，引进有实力的第三方物流公司，加快物流企业建设，增强发展实力。

繁荣现代服务业。充分发挥区位、交通、产业优势，建设改造一批商贸中心、综合批发等商贸物流市场，扶持发展一批以小商品及农副产品交易为主的商贸型中心集镇和专业市场，形成以大商场为龙头，商业街和专业市场为骨干，商业网点为基础的大商贸格局。加快通讯、信息、中介服务等产业发展，带动商贸、交通运输不断繁荣。积极发展金融保险业，拓展金融保险市场。优化信贷结构，加大小额农村信贷投入，支持地方经济发展。大力发展社区服务业，提供便民服务。

六、着力缩小山川差距，在山川协调发展上取得新突破

合理配置自然和社会资源，促进山川共济。加大扶贫开发力度，围绕硒砂瓜、枸杞、马铃薯、饲草、枣瓜间作、小茴香等山区特色产业，整合各类扶贫项目资金，实行捆绑式投入，确保扶贫开发项目建设成效。优化帮扶工作机制，强化责任考核，确保社会帮扶取得实效。发展职业教育，加强农业实用技术培训和职业技能培训，努力提高山区劳动力综合素质。发展“异地工业”，增强海原经济发展能力。大力发展劳务经济，确保山区每户常年输出一名劳动力。按照优先开发、重点开发、限制开发和禁止开发的不同要求，明确不同区域的功能定位，对不适于生存的乡村贫困人口实行移民搬迁。大力实施以“退人还山”为重点的山川协调发展和生态恢复工程。探索整村整乡、分散安置的移民搬迁新路子，改善贫困人口生存条件和人文环境。

七、着力建设先进文化，在文明中卫建设上取得新突破

培育文明风尚。认真贯彻《公民道德建设实施纲要》，在全社会大力弘扬爱国主义、集体主义、社会主义思想。加强社会公德、职业道德、家庭美德、个人品德建设，发挥道德模范榜样作用，引导人们自觉履行法定义务、社会责任、家庭责任。加强和改进思想政治工作，注重人文关怀和心理疏导，用正确的方式处理人际关系。动员社会各方面共同做好青少年思想道德教育工作，为青少年健康成长创造良好的社会环境。深入开展群众性精神文明、诚信中卫创建活动。大力倡导男女平等、尊老爱幼、互爱互助、见义勇为的社会风尚。弘扬科学精神，普及科学知识，提高全民科技素质。

办好人民满意的教育。坚持把教育摆在优先发展的战略地位，教育投入年均增长25%。优化教育结构，形成各级各类教育全面协调可持续发展的良好格局。全面加强基础教育，重视学前教育，关心特殊教育，促进教育公平。巩固和提升“普九”成果，初中毛入学率达到98%。扩大高中办学规模，普及高中阶段教育。积极办好公办职业院校，支持发展民办职业教育。建成南华山职业技术学校和中卫职业技术学院，力争使所有初、高中毕业不能升学者都能进入职业技术学校培训，为社会输送技能人才和创业人才。加强教师和校长队伍建设，全面提高教学质量和水平。规范教育收费，建立健全资助制度，保证经济困难家庭、进城务工人员子女平等接受义务教育。

建设先进文化。完善扶持公益性文化事业、文化产业发展和文化创新政策，营造有利于出精品、出人才、出效益的环境，提高公共文化产品的生产供给能力。抓好历史文化遗址和非物质文化遗产保护。组建中卫市专业文艺团体，鼓励发展民间文艺团体。重视城乡、区域文化协调发展。加强网络文化建设和管理，营造良好网络环境。加强体育基础设施建设，广泛开展全民健身活动。提高竞技体育水平。

八、着力改善人民生活，在促进社会和谐上取得新突破

促进以创业带动就业，努力让群众劳有所得。实行积极的就业政策，用发展的办法解决就业问题。统筹做好城镇新增劳动力就业、农村富余劳动力转移就业、下岗失业人员再就业工作。切实加强对大学毕业生、退役军人就业指导和服务。完善和落实国家对农民工的保护政策。规范发展就业服务机构，建设城乡统一的劳动力市场。开展全民创业活动，设立青年创业基金，鼓励自主创业，支持自谋职业，使更多劳动者成为创业者。积极争取公益性岗位，及时帮助就业困难人员就业。

完善城乡社会保障体系，努力让群众老有所养。加强基金监管，实现保值增值。推进机关、事业单位养老保险制度改革，完善企业职工基本养老保险和城乡低保制度，逐步提高城乡居民低保水平。推进失业、工伤、生育保险制度建设。开展农村养老保险制度试点。整合救助资源和救助资金，完善农村五保户供养、特困户救助、灾民救助等制度。发展社会福利和慈善事业。完善住房公积金制度和廉租房制度，着力解决城镇低收入家庭和特殊困难家庭住房问题。严格执行计划生育基本国策，促进人口与经济、社会、环境资源协调可持续发展。

加强医疗卫生保障，努力让群众病有所医。完善重大疾病防控体系，做好重大疾病和传染病防治工作，提高突发公共卫生事件的应急处置能力。合理布局社区卫生服务机构，加快推进城乡医疗卫生机构标准化建设。积极推行新型农村合作医疗，建立以大病统筹为主的城乡居民基本医疗保险和救助制度，做到全民医保。坚持药招“三统一”，保证群众用上廉价药品。开展爱国卫生运动。发展妇幼卫生事业。

维护社会安定团结，努力让群众居有所安。加强劳动保护，维护广大职工特别是进城务工人员的合法利益。做好残疾人、老龄工作。维护妇女儿童合法权益。加强社区建设，完善基层自治组织，扩大基层民主。完善社会突发事件应急管理体制机制，增强应对公共突发事件的能力。强化安全生产管理和监督，预防和减少各类安全事故的发生。深化产品质量和食品药品安全专项整治，保证人民群众饮食、用药安全。加强环境保护和防震减灾工作，促进人与自然和谐共处。认真开展“五五普法”和平安中卫创建活动，加强社会治安综合治理，维护社会安定团结。增强全民国防观念，加强国防动员建设，创建双拥模范城。全面贯彻党的民族宗教政策，依法加强对宗教事务的管理，巩固和发展平等团结、和谐互助的社会主义民族关系。

九、着力推进政府自身建设，在全面提高政府行政效能上取得新突破

加强学习，提高依法行政质量。大力倡导学习之风，不断加强公务人员的理论武装、知识更新和能力培养。汇编行政法律法规，实施公务员职业化培训五年规划，从2008年开始，对全市公务员进行政治理论、市场经济、科技法律和职业技能培训，彻底解决部分公务员不懂岗位专业，不掌握行政法律、法规和政策，不熟悉经济工作等问题，全面提高公务员依法行政的自觉意识和解决实际问题的能力，培养一支具有执行力的高素质公务员队伍。

健全制度，提高依法行政效率。完善市长信箱制度，直接受理群众来信来电和来访，直接接受群众的监督，让行政阳光化。坚持政府领导和部门负责人每周接访等制度，一件不搁地解决好人民群众的合理要求和困难。完善会议制度，少开总结表彰会，多开研究解决问题的办公会、专题会，让领导和干部从不解决问题的会议中解脱出来。设立政务大厅，建立“一站、限时”政务办理流程和首问责任制。建立“偿贷基金”，为政府借钱发展搭建融资平台，为防范财政风险提供财力担保。实行部门编制增减动态管理，对工作量少、编制岗位多的部门减编，对工作量大、编制岗位少的部门增编，优化编制配置，提高行政效率。建立市长督办副市长、市长助理和重点部门制度，形成一级督办一级、一级对一级负责的执行落实机制。把督办结果列入年度综合考评之中，切实提高政府执行力。

强化监督，提高依法行政保障。自觉接受人民代表大会及其常务委员会的依法监督和人民政协的民主监督，认真办理人大议案、代表建议和政协委员提案。积极支持法院、检察院依法办案。重视和发挥工会、共青团等人民团体的桥梁纽带作用，加强同各民主党派、工商联、无党派人士的联系，虚心听取各界人士的意见和建议，使各项决策符合法律法规,符合实际情况，符合人民心愿。

防惩并重，筑牢廉洁从政基础。认真落实领导干部廉洁自律规定和党风廉政建设责任制，突出抓好各级干部特别是领导干部的廉洁自律，坚决查处违纪违法案件，坚决纠正损害人民群众利益的不正之风，建立健全教育、制度、监督并重的惩治和预防腐败工作体系。加强审计监督，严肃财经纪律，强化对权力的制约与监督。坚持“两个务必”，反对铺张浪费、贪污腐化，树立廉洁从政的良好形象。

2008年重点工作

各位代表，2008年是全面贯彻落实党的十七大精神的第一年，也是本届政府的开局之年。做好2008年的工作意义重大。全市经济社会发展主要预期目标是：地区生产总值突破100亿元大关，增长13%以上；地方财政一般预算收入4.1亿元，可比增长13.2%；全社会固定资产投资82.5亿元，增长15%；社会消费品零售总额22.3亿元，增长17%；城镇居民人均可支配收入10400元，增长12.4%；农民人均纯收入3380元，增长10.5%；万元地区生产总值综合能耗降低4%；人口自然增长率控制在12.5‰以内；城镇登记失业率控制在4.2%以内。重点工作是：

在工业方面，完成美利工业区起步区水、电、路、污水处理厂、供水厂、两个水库、一个生化氧化塘等基础设施建设和道路两侧绿化工作。争取入园企业达到5家，到位资金5亿元。重点抓好中冶美利纸业林纸一体化二期、中电投2×33万千伏热电、江苏扬农一体化项目、宁夏红集团枸杞饮料、宁夏美康陶瓷地砖、宁夏隆基单晶硅、天元公司金属锰等项目。在农业方面，加快发展硒砂瓜、设施蔬菜、枸杞等优势特色农业，做好硒砂瓜的种子供应和市场营销工作，确保种得好、卖得出、效益高。硒砂瓜种植面积达到98万亩以上，新增设施蔬菜4万亩，发展优质苹果8万亩，枸杞2万亩，完成4万亩生态经济林带建设。规划建设镇罗蔬菜批发市场。在城市建设方面，完成行政中心和行政广场、香山公园及新区次干道路建设。完成新区和老区绿化改造工程。开工建设黄河公园二期工程，完成景观水道、黄河公园一期后续工程。开工建设第二人民医院。完成中卫至中宁滨河大道建设。抓好中宁县集污及污水处理厂续建、南河子公园扩建和海原县牌路山森林公园等项目建设。在第三产业方面，完成沙坡头旅游公司改制和重组工作。修编沙坡头旅游区、沙漠湿地旅游区等重点景区控制性详规。规划建设沙坡头至黄河公园旅游观光带和城市森林公园，发展壮大旅游产业。办好奥运火炬中卫站传递活动。建立硒砂瓜百里观光长廊。积极发展现代物流业，研究制定发展规划和配套政策，规划建设中卫物流园区。在山川协调发展方面，配合自治区做好海原县行政区划调整和党政机关迁建工作。规划建设生态移民区，整体搬迁4个村、15个自然村5176人。其中海原县搬迁3603人。在教育方面，建成南华山职业技术学校、中卫第六中学和中卫第八小学。在民生事业方面，建立大病统筹救助、失业人员和家庭困难人员救助制度，加强社会保障和社会救助，解决部分困难群众的基本保障问题，建成社区医疗服务体系和社会福利院。在重点基础设施方面，建成沙坡头机场。在文化体育建设方面，开工建设市体育馆、老干部活动中心等一批文化体育设施。在体制机制创新方面，出台职业教育、中小企业贷款贴息办法、担保办法，风险投资制度、小额贷款办法、农业生产设施所有权改革、行政效能建设等制度和政策。

各位代表！中卫跨越式发展的进军号角已经吹响。让我们在市委的正确领导下，紧紧依靠全市人民，以百折不挠的意志、心无旁骛的信念、全力以赴的投入，在跨越式发展的征程中攻坚克难、建功立业，为建设一个和谐并进、充满生机、魅力四射的中卫而努力奋斗。
